# IGFBP2 drives epithelial-mesenchymal transition in hepatocellular carcinoma via activating the Wnt/β-catenin pathway

**DOI:** 10.1186/s13027-023-00543-6

**Published:** 2023-11-13

**Authors:** Xiu Chen, Yu Zhang, Pingping Zhang, Mengzhu Wei, Tian Tian, Yanling Guan, Chenchen Han, Wei Wei, Yang Ma

**Affiliations:** Institute of Clinical Pharmacology, Key Laboratory of Anti-Inflammatory and Immune Medicine, Ministry of Education, Anhui Collaborative Innovation Center of Anti-Inflammatory and Immune Medicine, Center of Rheumatoid Arthritis of Anhui Medical University, Anhui Medical University, Hefei, 230032 China

**Keywords:** Hepatocellular carcinoma, Epithelial-mesenchymal transition, IGFBP2, β-catenin, LRP6

## Abstract

Metastasis has emerged as a major impediment to achieve successful therapeutic outcomes in hepatocellular carcinoma (HCC). Nonetheless, the intricate molecular mechanisms governing the progression of HCC remain elusive. Herein, we present evidence highlighting the influence exerted by insulin-like growth factor-binding protein 2 (IGFBP2) as a potent oncogene driving the malignant phenotype. Our investigation reveals a marked elevation of IGFBP2 expression in primary tumors, concomitant with the presence of mesenchymal biomarkers in HCC. Through in vitro* and *in vivo experimentation, we demonstrate that the overexpression of IGFBP2 expedites the progression of epithelial-mesenchymal transition (EMT) and facilitates the metastatic potential of HCC cells, chiefly mediated by the Wnt/β-catenin signaling pathway. Notably, knockdown of IGFBP2 significantly decreased the expression of total and nuclear β-catenin, N-cadherin and vimentin in the treatment of the specific activator of Wnt/β-catenin CHIR-99021. Collectively, our findings identify IGFBP2 as a pivotal regulator within the HCC EMT axis, whereby its overexpression confers the distinctly aggressive clinical features characteristic of the disease.

**Supplementary Information:**

The online version contains supplementary material available at 10.1186/s13027-023-00543-6.

## Introduction

Hepatocellular carcinoma (HCC) ranks as the third leading cause of cancer-related mortality worldwide, with a grim 5-year survival rate of approximately 18% [1]. Chronic hepatitis B can progressively lead to liver cirrhosis, which is an independent risk factor for HCC, about 14% of people infected with the virus die from HCC each year. The disease claims around 830,000 lives annually, underscoring its severe prognosis [1]. The prevalence of intra-hepatic and extrahepatic metastasis contributes significantly to the unfavorable clinical outcomes observed in HCC patients [2]. Consequently, the identification and management of metastatic tumors pose the greatest hurdle in HCC clinical trials.

Epithelial–mesenchymal transition (EMT), a crucial event in metastasis, plays a pivotal role in the metastatic progression of hepatocellular carcinoma (HCC). During the invasion of solid tumors into adjacent cell layers, tumor cells undergo EMT, thus resulting in the loss of cell adhesion and the acquisition of motility. Various transcription factors, such as Slug, Snail, Twist, and Zeb families [3], orchestrate EMT, endowing cancer cells with metastatic properties by enhancing their mobility, invasiveness, and resistance. Therefore, investigating EMT suppressors and elucidating the underlying mechanisms hold therapeutic potential for HCC.

Active β-catenin directly binds to the promoters of transcription factors associated with key EMT inducers, including Slug, Twist, and Zeb1, thereby inducing their expression [4]. β-catenin serves as the key effector in the canonical Wnt pathway, by transmitting signals to the nucleus [5]. The low-density lipoprotein receptor-associated protein 6 (LRP6) functions as an essential coreceptor in Wnt/β-catenin signaling. CHIR-99021 is a potent and selective GSK-3α/β inhibitor and the Wnt/β-catenin signaling pathway activator, stabilizes β-catenin and activates Wnt/β-catenin pathway [6]. Upon Wnt activation, LRP6 undergoes phosphorylation and interacts with axis inhibitor (AXIN) and GSK-3β, preventing the phosphorylation and subsequent degradation of β-catenin [7]. Consequently, nonphosphorylated β-catenin accumulates in the cytoplasm and translocates into the nucleus, where it associates with the T-cell factor/lymphoid enhancer factor 1 (TCF/LEF1) transcription complex to facilitate the transcription of target genes [8, 9].

IGFBPs is a class of secreted proteins that is able to interact with many ligands including IGFs, its member includes IGFBP1-7. The anti-tumoral effect of lGFBP-3 is due to inhibition of the Wnt pathway through CD44, a receptor protein known to modulate Wnt signaling. IGFBP7 promotes gastric cancer by enhancing TAM/M2 macrophage polarization through FGF2/FGFR1/PI3K/AKT axis. IGFBP7 in circulation acts as a biomarker and key mediator of acute kidney injury by inhibiting RNF4/PARP1-mediated tubular injury and inflammation [10–12]. As the second most abundant insulin-like growth factor-binding protein (IGFBP) in human circulation. IGFBP2 exhibits a profound affinity for IGFs, rendering it a key player in the intricate network of biological interactions. Notably, elevated levels of IGFBP2 have been consistently observed in both the plasma [13, 14] and tumor cells [15, 16] of many patients with malignant tumors. Previously we showed IGFBP2 is elevated in the plasma of HCC patients and pedicts poor prognosis [17, 18]. Nonetheless, the precise mechanisms by which IGFBP2 governs the process of epithelial-mesenchymal transition (EMT) in HCC cells remain unclear, necessitating further exploration and elucidation.

In the present study, we put forth the hypothesis that IGFBP2 serves as an instigator in HCC and that this induction of EMT is mediated via activation of the Wnt/β-catenin pathway. To validate the hypothesis, we conducted investigations using an in vivo mouse model, clinical samples and HCC cell lines in vitro.

## Materials and methods

### Human tissue specimens and immunohistochemical analysis

Under the approval of the Biomedical Ethics Committee of Anhui Medical University (No. 20150296, Hefei, China), we obtained 50 tumor samples from HCC patients who received treatment at the hospital between 2010 and 2015. These patients underwent hepatectomy without preoperative radiotherapy or chemotherapy. Two pathologists confirmed the HCC diagnosis for all 50 patients who underwent surgery. For immunohistochemical (IHC) analysis, consecutive sections of formalin-fixed, paraffin-embedded tumors were processed following the manufacturer's instructions using a DAB substrate kit. IGFBP2, E-cadherin, N-cadherin, and vimentin were assessed, and the scoring was conducted by two pathologists who were blinded to the clinicopathologic data. We examined the IHC with different cohort of HCC patients and analyzed the correlation between IGFBP2 expression and clinical features.

### Cell culture and reagents

HepG2, a human HCC cell line, was obtained from ATCC, while HCCLM3 and SMMC-7721 were obtained from the Shanghai Cell Bank, Chinese Academy of Sciences. All cell lines were cultured in Dulbecco's modified essential medium (DMEM) supplemented with 10% fetal bovine serum (FBS). The cells were incubated at 37 °C in a humidified chamber with 5% CO_2_ to provide optimal growth conditions.

### SiRNA, plasmid construction, and transfection in HCC cells

The pcDNA3.1 plasmid was utilized as the vector for cloning the full-length human IGFBP2 cDNA, which was subsequently validated through sequencing. The resulting construct was then transfected into HepG2 cells to establish stable expression of IGFBP2. Genepharma designed specific small interfering RNAs (siRNAs) targeting IGFBP2 for the purpose of targeted gene silencing. For the transfection procedure, cells at 80% confluence were seeded in 6-well plates at a density of 5 × 10^5^ cells/well in medium supplemented with 10% FBS. Once the cells reached 80% confluence, they were transfected with either 2 μg of plasmids or 50 nM of siRNAs using Lipofectamine 2000 (Invitrogen) for 48 h. To facilitate the selection of stably transfected cells, G418 treatment was applied.

### Western blot analysis

The cells were lysed in RIPA buffer supplemented with a proteinase inhibitor cocktail to prepare whole-cell extracts. The nuclear and cytoplasmic components were separated using the NE-PER Nuclear and Cytoplasmic Extraction Reagents kit (Thermo Fisher Scientific), following the manufacturer's protocol. Twenty micrograms of lysates mixed with SDS-loading buffer were separated by SDS-PAGE and transferred to PVDF membranes. The membranes were blocked in PBS containing 5% BSA at 4 °C overnight, followed by a 1 h incubation with the following primary antibodies: anti-IGFBP2 rabbit polyclonal antiserum (1:1000 dilution, Cell Signaling), anti-β-catenin (1:1000 dilution, Cell Signaling), anti-E-cadherin, N-cadherin, and Vimentin (1:1000 dilution, Proteintech), and β-actin (1:2000 dilution, Santa Cruz). Subsequently, the membranes were incubated for 30 min with horseradish peroxidase-conjugated anti-rabbit or anti-mouse IgG antibodies (1:10,000 dilution, Beijing Zhong Shan-Golden Bridge). The specific proteins were detected using an enhanced chemiluminescence Western blotting analysis system, and the target proteins were visualized by Western blotting.

### Immunofluorescence imaging and immunohistochemical staining

The tumor samples underwent deparaffinization in xylene followed by rehydration in ethanol. Immunostaining and imaging procedures were performed following previously established protocols [19]. Cell suspensions were aseptically seeded onto 6-well plates. After transfection with plasmids or siRNA for 48 h, the cells were treated with 4% paraformaldehyde and 0.25% Triton X-100 at room temperature. Subsequently, the cells were incubated with specific antibodies targeting E-cadherin, N-cadherin, and Vimentin. DAPI staining was employed to visualize nuclear localization, and laser scanning confocal microscopy was used for examination. All images were captured using an optical microscope.

### Quantitative real-time RT-PCR

Total RNA was extracted from the samples using an RNAsimple Total RNA Kit (Tiangen). Subsequently, 1 µg of total RNA was reverse transcribed into cDNA using the Prime Script RT reagent Kit (TaKaRa). Real-time PCR analysis was conducted using the SYBR Green PCR Kit (TaKaRa) with specific primers targeting IGFBP2, N-cadherin, E-cadherin, Vimentin, Slug, and Snail. The mRNA expression levels were normalized to GAPDH mRNA levels.

### Migration and invasion assays

Invasion assays were conducted using matrigel-coated transwell chambers with an 8.0 μm pore size (BD Biosciences). In each assay, 2 × 10^5^ HCC cells were seeded in the upper chamber, which contained serum-free DMEM. The lower chamber was filled with DMEM supplemented with 10% FBS, serving as a chemoattractant. The cells were allowed to invade for 24 h. Following the incubation period, the cells that successfully invaded through the filter into the lower chamber were stained using a 0.5% crystal violet solution (Sigma) and visualized. Each experiment was performed in triplicate, and the presented results represent the mean values obtained.

### Luciferase reporter assay

A total of 5 × 10^5^ HepG2 cells were seeded into 24-well plates and transfected with 0.8 µg of Super 8XTOPFLASH or Super8XFOPFLASH plasmids, along with 0.1 µg of pRL-TK, using Lipofectamine 3000 (Invitrogen). After 24 h of transfection, the transfected cells were stimulated with either 250 ng/mL of recombinant human IGFBP2 or 100 ng/mL of the Wnt/β-catenin signaling activator CHIR-99021 for a duration of 4 h. The TCF reporter activity was subsequently measured using the Dual-Luciferase Assay System (Promega).

### Co-immunoprecipitation assay (Co-IP)

HepG2 whole-cell lysates were prepared using a lysis buffer containing 25 mmol/L HEPES, 150 mmol/L NaCl, 5 mmol/L EDTA, 1% Triton X-100, 10% glycerin, phosphatase inhibitors, and protease inhibitors. The lysates were kept on ice for 30 min, and cellular debris were removed by centrifugation at 12,000*g* for 15 min at 4 °C. Prior to immunoprecipitation, the lysates were precleared using normal mouse IgG for 2 h at 4 °C. Simultaneously, LRP6 antibodies or corresponding IgG were added to protein A/G plus-agarose and incubated for 2 h at 4 °C. The precleared lysates were then incubated with the antibody-bead complexes overnight at 4 °C. The immune complexes bound to the beads were washed three times extensively using the lysis buffer. To denature the proteins, the lysates were mixed with SDS-PAGE loading buffer and heated for 10 min. Protein levels were assessed by standard SDS-PAGE and detected using LRP6 or IGFBP2 antibodies.

### Tumor xenograft model

Male Balb/c nude mice aged three to four weeks were obtained from Shanghai SLAC Laboratory Animal Co. Ltd (Shanghai). HepG2/pcDNA3.1-Ctr or HepG2/pcDNA3.1-IGFBP2 cells (1 × 10^6^) were mixed with Matrigel (BD Biosciences) and subcutaneously injected into the flank of each mouse, there were 5 mice in each group. Tumor growth was monitored for a period of 6 weeks following cell injection. All experimental procedures were conducted in accordance with the principles of laboratory animal care guidelines and were approved by the Biomedical Ethic Committee for Animal Experimentation of Anhui Medical University (No. 20150296, Hefei, China).

### Statistical analysis

The data are presented as mean ± standard deviation (SD) from a minimum of three independent experiments performed in triplicate. Statistical analysis was conducted using the Student's t-test for comparing two groups, while ANOVA was used to compare continuous variables across multiple groups. The correlations between the clinicopathological features and IGFBP2 staining scores were analyzed using the chi-square (χ^2^) test. Statistical analyses were performed using SPSS version 22.0 and GraphPad Prism 6.02 software.

## Results

### Overexpressed IGFBP2 is a driver of tumor metastasis in HCC

**Fig. 1:**
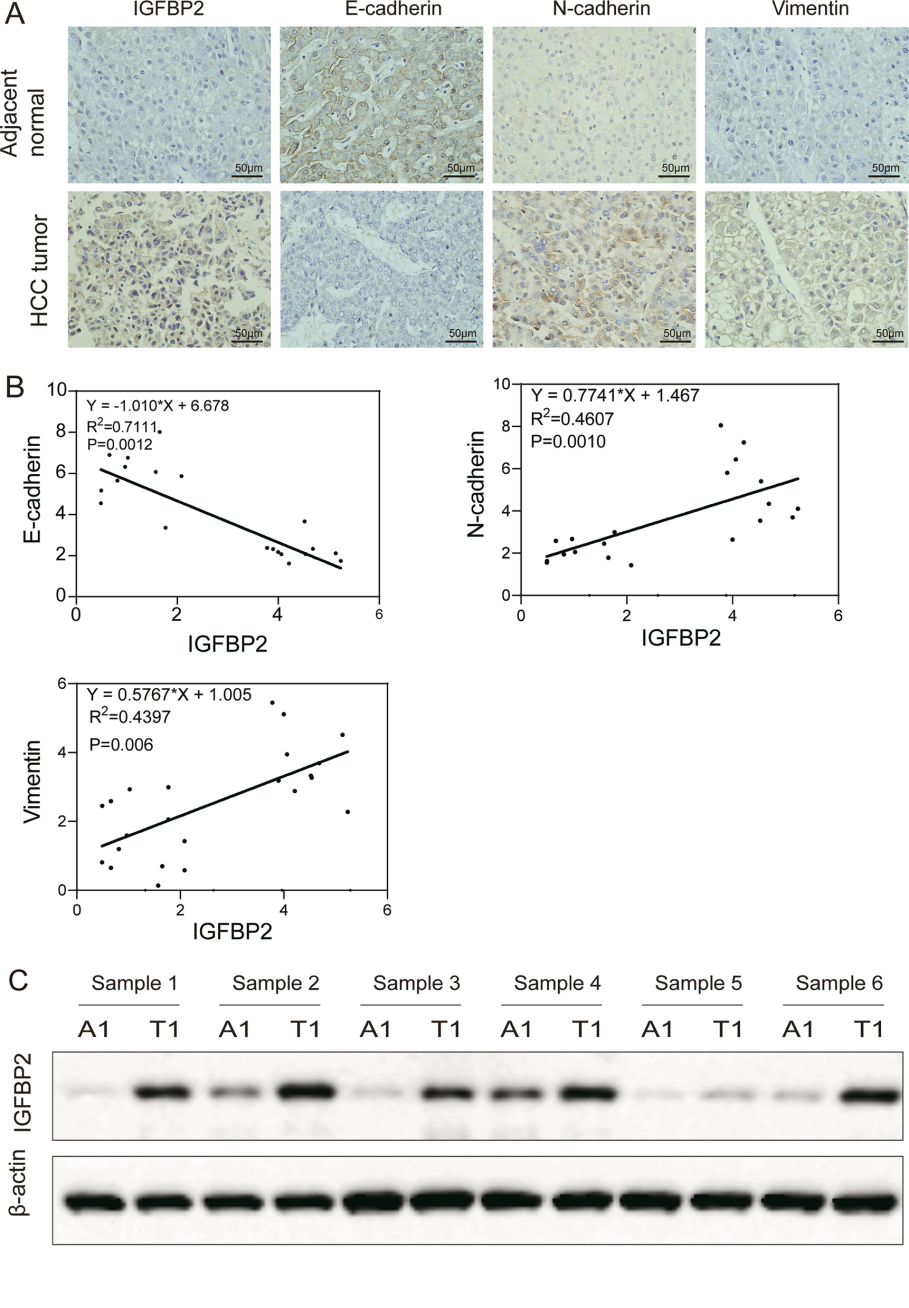
IGFBP2 is overexpressed in HCC tissues. **A** Expression of IGFBP2 and EMT related proteins in adjacent tissues and tumor tissues of HCC patients were detected by immunohistochemistry. Scale bars, 50 μm. **B** Correlation between the level of IGFBP2 and the expression of EMT related proteins. **C** Expression of IGFBP2 in adjacent tissues and tumor tissues of HCC patients was detected by Western blot.

Immunohistochemical analysis revealed robust expression of IGFBP2 in tumor tissues of HCC patients, whereas its expression was seldom detected in para-carcinoma tissues (Fig. 1A). Consistently, Western blot analysis demonstrated a significant upregulation of IGFBP2 expression in tumor tissue compared to adjacent tissue in five out of the six tested sample pairs (Fig. 1C, P < 0.001). Significant correlation was observed between the level of IGFBP2 and the expression of mesenchymal markers, including vimentin and E-cadherin (Fig. 1B). Furthermore, Based on the quantification of IHC signal intensities, the patients were divided into IGFBP2 low (−/+) and high (++/+++) expression groups, and the correlation between IGFBP2 expression and clinical pathology features was assessed. A high level of IGFBP2 was positively correlated with tumor size, and organ metastasis (Additional file 1: Table S1). These findings strongly suggest that the overexpression of IGFBP2 in HCC correlates with tumor metastasis.

### IGFBP2 promotes EMT in HCC cells

**Fig. 2:**
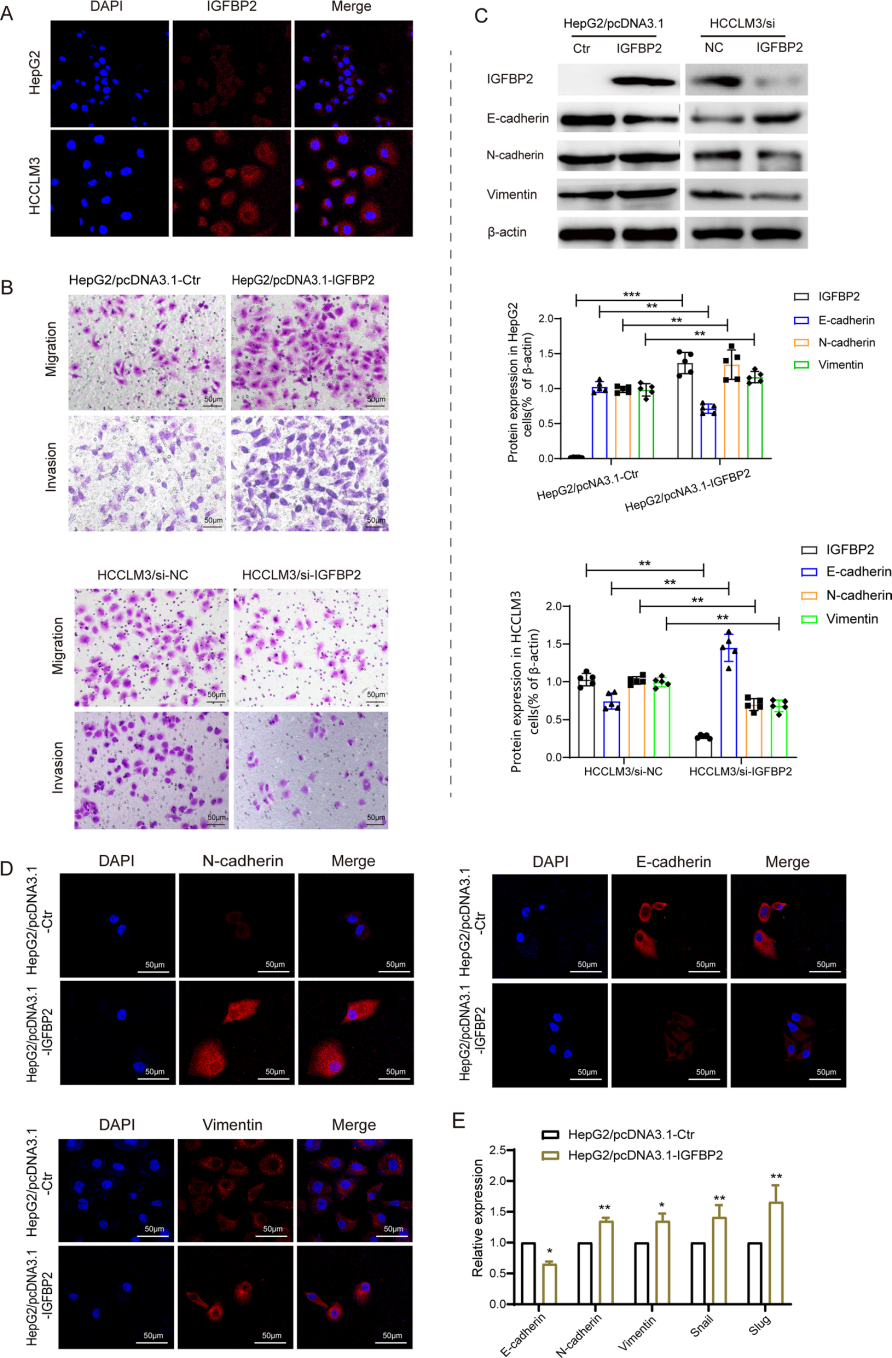
IGFBP2 drives EMT to promote invasion and metastasis in HCC cells. **A** Expression of IGFBP2 in HCC cells was detected by immunofluorescence. Scale bars, 50 μm. **B** Effect of IGFBP2 overexpression on the migration and invasion of HCC cells. Scale bars, 50 μm. **C** Effect of IGFBP2 on the expression of EMT related proteins in HCC cells was detected by western blot. **D** Effect of IGFBP2 on the expression of EMT related proteins in HCC cells was detected by immunofluorescence. Scale bars, 50 μm. **E** Effect of IGFBP2 on the expression of EMT related genes in HCC cell lines were detected by RT-PCR. *p < 0.05 versus HepG2/pcDNA3.1-Ctr, **p < 0.01 versus HepG2/pcDNA3.1-Ctr

The observed clinical association between IGFBP2 and mesenchymal markers prompted us to investigate the underlying molecular mechanisms regulated by IGFBP2 in HCC cells. Immunofluorescence staining of IGFBP2 in HCC cell lines revealed higher expression in HCCLM3 cells and lower expression in HepG2 cells (Fig. 2A). Based on these findings, we selected HepG2 cells for stable overexpression of IGFBP2 and HCCLM3 cells for IGFBP2 knockdown experiments. Transwell assays demonstrated that increased IGFBP2 expression significantly enhanced cell migration and invasion in HepG2 cells, while IGFBP2 knockdown resulted in decreased migration and invasion in HCCLM3 cells (Fig. 2B). Further Western blot and immunofluorescence analyses revealed that IGFBP2 overexpression in HepG2 cells led to a decrease in E-cadherin expression and an increase in N-cadherin and vimentin expression (Fig. 2C, D). Conversely, IGFBP2 knockdown in HCCLM3 cells resulted in increased E-cadherin expression and reduced N-cadherin and vimentin expression (Fig. 2C, D, Additional file 2: Fig. S1A). Consistent with these findings, RT-PCR analysis of EMT-related genes showed similar trends to the Western blot and immunofluorescence results (Fig. 2E, Additional file 2: Fig. S1B). Collectively, these results indicate that IGFBP2 promotes EMT in HCC cells.

### IGFBP2 activates the Wnt/β-catenin signaling pathway in HCC cells

**Fig. 3:**
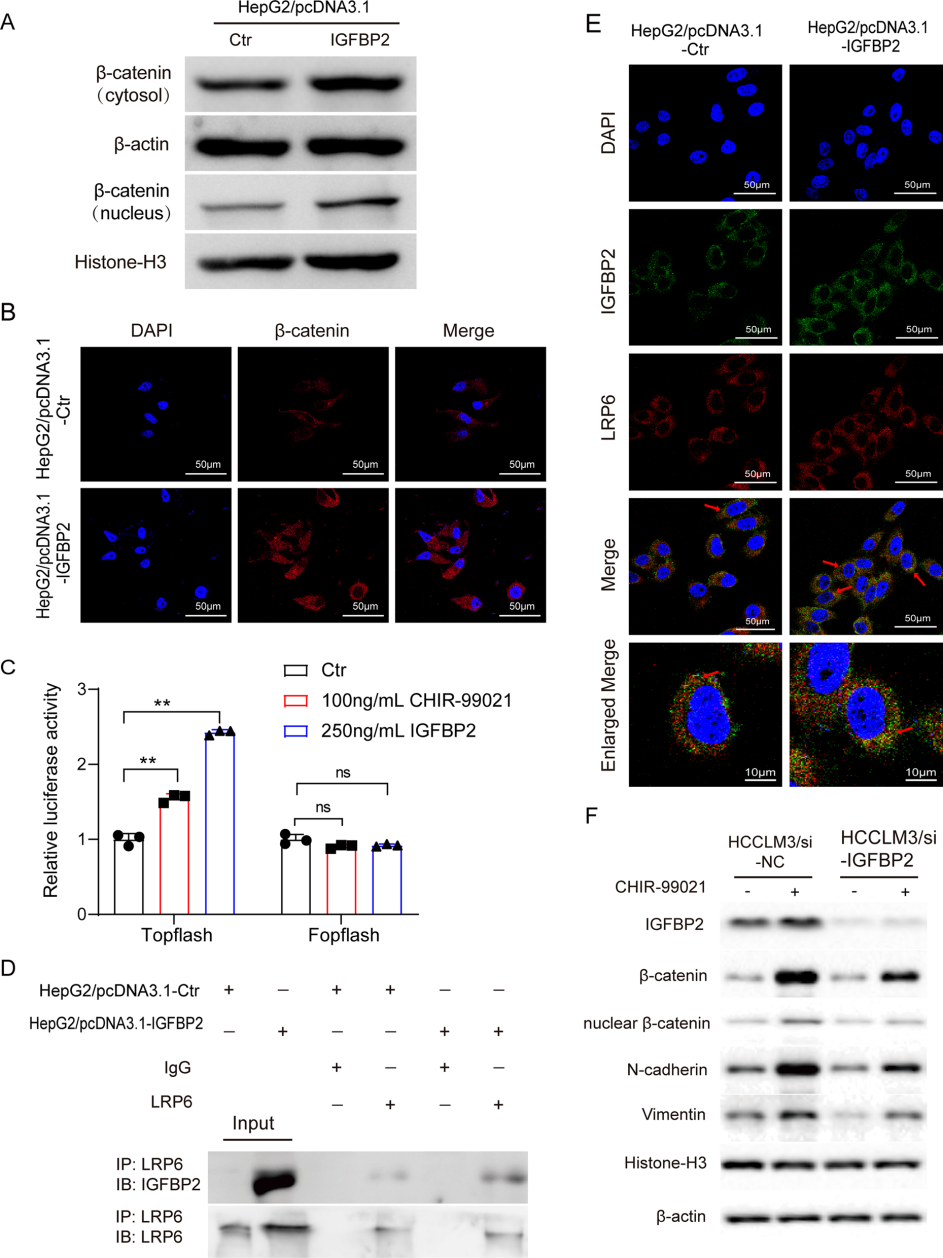
IGFBP2 drives EMT to promote invasion and metastasis in HCC cells via LRP6/ β-catenin signaling pathway. **A** Expression of β-catenin was detected by western blot. **B** Expression and localization of β-catenin was detected by immunofluorescence. Scale bars, 50 μm. **C** The transcript level of β-catenin stimulated by recombinant human IGFBP2 in HepG2 cells was detected by double luciferase reporter gene method. **p < 0.01 versus Ctr. **D** The interaction of LRP6 with IGFBP2 was detected by immunoprecipitation (CO-IP) after transfection of IGFBP2 plasmid or the vector control in HepG2 cells. **E** Co-expression of IGFBP2 and LRP6 were detected by immunofluorescence. Scale bars, 50 μm. **F** Expression of β-catenin and EMT related proteins were detected by western blot after CHIR-99021 treatment

As previously reported, the Wnt/β-catenin pathway plays a crucial role in EMT and cancer cell metastasis [20–22]. Therefore, we investigated whether IGFBP2 also regulates the canonical Wnt/β-catenin pathway. Overexpression of IGFBP2 in HepG2 cell lines resulted in increased levels of cytoplasmic β-catenin and enhanced nuclear accumulation of β-catenin (Fig. 3A). To further analyze the effect of IGFBP2 overexpression on β-catenin subcellular localization, we performed immunofluorescence staining, which confirmed that IGFBP2 overexpression promoted cytoplasmic and nuclear accumulation of β-catenin (Fig. 3B). Meanwhile, downregulation of IGFBP2 in HCCLM3 cell lines resulted in decreased levels of cytoplasmic and nuclear β-catenin (Additional file 3: Fig. S2A).

To further investigate the impact of IGFBP2 on the Wnt signaling pathway, we conducted a TOPFlash/FOPFlash system assay. The TOPFlash plasmid contains repeated LEF/TCF-binding motifs along with a luciferase gene, which serves as a LEF/TCF reporter, while the FOPFlash plasmid is a control with mutated TCF/LEF-binding motifs. Activation of the Wnt/β-catenin signaling pathway by CHIR-99021, a specific activator [23], leads to increased β-catenin-mediated LEF/TCF transcriptional activity. In accordance with these findings, stimulation with IGFBP2 significantly enhanced LEF/TCF transcriptional activity both in HepG2 and HCCLM3 cells. Conversely, the activity of FOPFlash remained unaffected by both IGFBP2 and CHIR-99021 treatment (Fig. 3C). These observations suggest that IGFBP2 promotes nuclear translocation of β-catenin and upregulates the transcriptional activity of LEF/TCF in HCC cells.

A previous study has demonstrated that IGFBP4 physically interacts with the Wnt receptor Frizzled 8 (Frz8) and the Wnt co-receptor low-density lipoprotein receptor-related protein 6 (LRP6) through the carboxy-terminal thyroglobulin domain [24]. IGFBP4 and IGFBP2 proteins, with a mass of ~ 24 to 50 kDa (216–289 amino acids), share a highly conserved structure with three domains of similar sizes: the conserved N-terminal cysteine rich region and the C-terminal cysteine rich region connected by a less structural and less conserved linker region [25]. Given that IGFBP2 shares the same domain with IGFBP4, we investigated whether IGFBP2 could also interact with Frz8 or LRP6. Co-immunoprecipitation (Co-IP) assays were performed to explore the interaction between endogenous LRP6 and IGFBP2. As shown in Fig. 3D, we found that LRP6 co-immunoprecipitated with IGFBP2 in HepG2 control cells, and this interaction was increased in HepG2 cells overexpressing IGFBP2. Immunofluorescence staining of IGFBP2 and LRP6 in HepG2 cells also supported these findings (Fig. 3E). These results suggest that IGFBP2 enhances β-catenin transcriptional activity, likely by interacting with the Wnt co-receptor LRP6.

### IGFBP2 induces EMT through Wnt/β-catenin signaling in HCC cell

To investigate the role of the Wnt/β-catenin pathway in IGFBP2 promoting HCC EMT, we examined the effects of specific activator of Wnt/β-catenin signaling on the expression of mesenchymal markers in HCCLM3/si-NC and HCCLM3/si-IGFBP2 cells (Fig. 3F). Our results showed that treatment with CHIR-99021 resulted in increased accumulation of total and nuclear β-catenin in HCCLM3/si-NC, as well as the increased expression of mesenchymal markers N-cadherin and vimentin compared with vehicle control, while knockdown of IGFBP2 significantly decreased the expression of total and nuclear β-catenin, N-cadherin and vimentin in the treatment of CHIR-99021 in HCCLM3/si-IGFBP2 cells compared with HCCLM3/si-NC (Fig. 3F, G), suggesting that knockdown of IGFBP2 inhibits the activation of β-catenin signaling and expression of mesenchymal markers. These findings implied that the Wnt/β-catenin signaling pathway may act as a downstream mediator of IGFBP2 controlling EMT in human HCC cell lines.

### IGFBP2 strengthens HCC tumorigenic ability in vivo

**Fig. 4:**
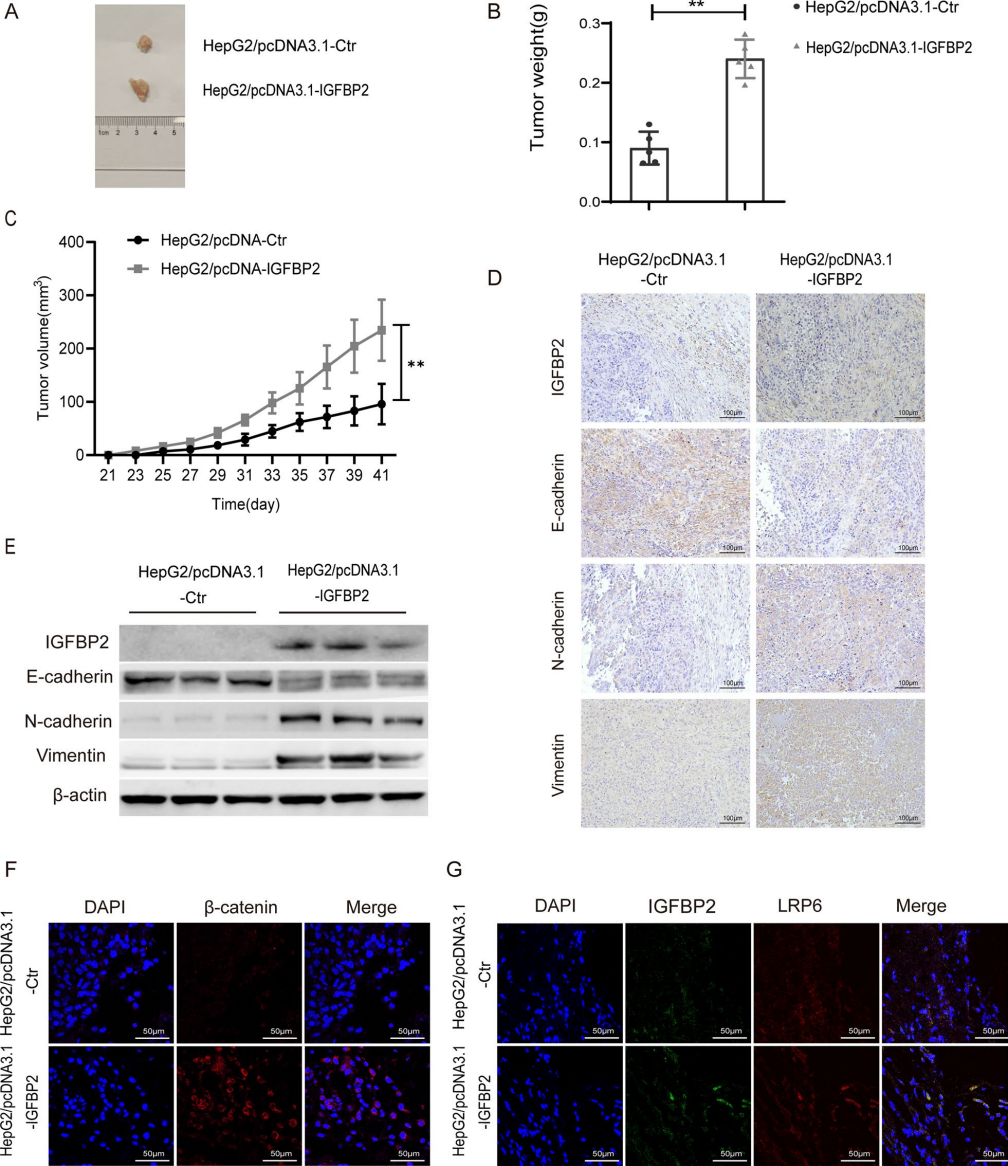
IGFBP2 strengthens HCC tumorigenic ability in vivo. **A** The figure of subcutaneous tumor tissue of nude mice. **B** The weight of subcutaneous tumor tissue of nude mice. **p < 0.01 versus HepG2/pcDNA3.1-Ctr. **C** The changes of tumor volume of nude mice. **p < 0.01 versus HepG2/pcDNA3.1-Ctr. **D** Expression of IGFBP2 and EMT related proteins were detected by immunohistochemistry. Scale bars, 100 μm. **E** Expression of IGFBP2 and EMT related proteins were detected by western blot. **F** Expression and localization of β-catenin in tumor tissues of nude mice was detected by immunofluorescence. Scale bars, 50 μm. **G** Co-expression of IGFBP2 and LRP6 in tumor tissues of nude mice were detected by immunofluorescence. Scale bars, 50 μm

To further validate our in vitro findings, we investigated the role of IGFBP2 in tumorigenic ability in vivo. Tumor xenograft experiments were conducted using HepG2 cells with IGFBP2 overexpression and with vector control, and the results showed enhanced tumor progression in the group with IGFBP2 overexpression compared with the control group, as evidenced by the increased tumor weight and volume (Fig. 4A–C). Subsequent Western blot and immunohistochemistry analyses of the tumor tissues revealed lower expression of E-cadherin and higher expression of N-cadherin and vimentin in response to IGFBP2 overexpression (Fig. 4D, E). Moreover, IGFBP2 overexpression led to increased accumulation of β-catenin in both the cytoplasm and nucleus (Fig. 4F). Additionally, immunofluorescence assay demonstrated an enhanced co-localization of IGFBP2 and LRP6 in the mouse tumor tissues (Fig. 4G). Collectively, these findings indicate that IGFBP2 enhances the tumorigenic ability of HCC in vivo.

## Discussion

**Fig. 5:**
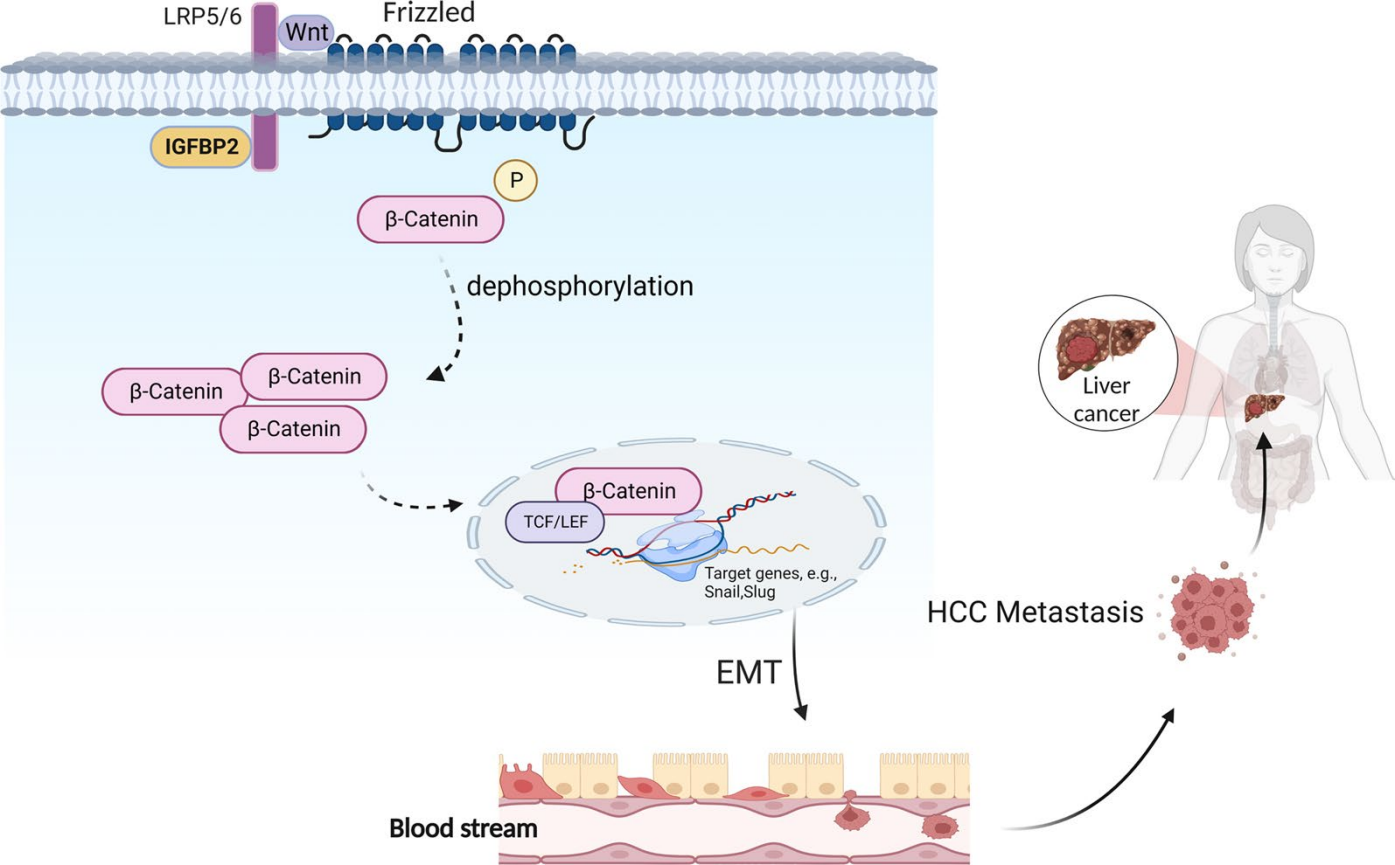
The mechanism of IGFBP2 promoting invasion and metastasis of HCC. IGFBP2 interacts with LRP6, and then β-catenin is dephosphorylated. Nonphosphorylated β-catenin accumulates in the cytoplasm and is translocated into the nucleus where it interacts with the TCF/LEF1 transcription complex to promote target gene transcription. Gene expression causes the occurrence of EMT phenomenon which eventually leads to the invasion and metastasis of HCC

While the oncogenic role of IGFBP2 has been established in various tumor types, including breast, ovarian, glioma and prostate tumors [13, 14, 26], its specific mechanisms and involvement in HCC have remained elusive. In this study, we provide evidence that IGFBP2 is significantly overexpressed in HCC tumor tissues and its expression levels correlate with several mesenchymal biomarkers, while exhibiting a negative association with epithelial markers. Importantly, previous reports have shown that IGFBP2 overexpression predicts a poor prognosis and serves as an independent prognostic indicator for both time to recurrence and overall survival in HCC patients [18]. Therefore, our findings not only contribute to a deeper understanding of the role of IGFBP2 in HCC but also have the potential to serve as a valuable tool for predicting metastasis risk in HCC patients with high IGFBP2 expression levels (Fig. 5).

EMT is a critical cellular process that drives metastasis and invasion in HCC [27–29]. Previous studies have implicated various growth factors, including TGFβ1, in triggering EMT [30]. However, the role of TGFβ1 in HCC is complex, as it can also exhibit tumor suppressor functions [31]. Therefore, targeting TGFβ1 as a therapeutic strategy for HCC remains uncertain. In our study, we have identified IGFBP2 as a novel inducer of EMT in HCC. Overexpression of IGFBP2 in HepG2 cells induced EMT, while knockdown of IGFBP2 in HCCLM3 cells reversed this process. These findings suggest that inhibition of IGFBP2 expression may represent a promising approach for counteracting metastasis in HCC, offering a potential therapeutic avenue to explore.

Our study revealed that the induction of EMT by IGFBP2 is mediated through the activation of the Wnt/β-catenin pathway. The canonical Wnt pathway has been established as a crucial regulator of the EMT process in various contexts [32]. Activation of Wnt/β-catenin signaling leads to the stimulation of several EMT-related transcription factors, including Slug, Snail, Twist, ZEB1, and ZEB2. In our study, we showed that Wnt/β-catenin signaling pathway may act as a downstream mediator of IGFBP2 controlling EMT. We observed that treatment with CHIR-99021 resulted in increased accumulation of total and nuclear β-catenin in HCCLM3/si-NC, as well as the increased expression of mesenchymal markers N-cadherin and vimentin compared with vehicle control, while knockdown of IGFBP2 significantly decreased the expression of total and nuclear β-catenin, N-cadherin and vimentin in the treatment of CHIR-99021 in HCCLM3/si-IGFBP2 cells compared with HCCLM3/si-NC, demonstrating that knockdown of IGFBP2 significantly decreased the expression of total and nuclear β-catenin, N-cadherin and vimentin in the treatment of the specific activator of Wnt/β-catenin CHIR-99021. Furthermore, our study demonstrated that IGFBP2 physically interacts with the Wnt co-receptor LRP6, and this interaction is strengthened in cells overexpressing IGFBP2. These findings suggest that IGFBP2 promotes β-catenin activity, potentially through its interaction with the Wnt co-receptor LRP6, highlighting the role of IGFBP2 in modulating the Wnt/β-catenin signaling pathway during EMT in HCC.

Recent research demonstrated that IGFBP2 activates integrin β1 pathways, recruits ILK for cell migration, and activates NF-κB in glioma. Consistently, IGFBP2 promoted invasion and metastasis of pancreatic ductal adenocarcinoma by PI3K/Akt/IKKβ/NF-κB pathway [24], Notably, publication showed that IGFBP2 knockdown resulted in significant changes in the expression of genes associated with several pathways, cell cycle, p53 and Wnt pathways. So Wnt/β-catenin may not be the only oncogenic pathway linked to IGFBP2 in HCC, further study needs to investigate the other pathways underlying HCC metastasis.

In conclusion, our comprehensive investigations of IGFBP2 expression and its functional role in HCC patients, HCC cell lines, and nude mouse tumor xenograft models have provided evidence supporting the involvement of IGFBP2 in the induction of EMT through activation of the Wnt/β-catenin signaling pathway. These findings position IGFBP2 as a promising therapeutic target and a valuable biomarker for predicting metastatic risk and assessing prognosis in HCC.

### Supplementary Information

**Additional file 1**. Correlation between the IGFBP2 expression and the clinicopathologic features of HCC. Statistical analyses were carried out using Chi-square χ2 test , * represented p < 0.05 was considered significant.

**Additional file 2: Fig. S1**. Effect of IGFBP2 on the expression of EMT related proteins in HCCLM3 cell were detected by immunofluorescence. Scale bars, 50 μm. (B) Effect of IGFBP2 on the expression of EMT related gens in HCCLM3 cell were detected by RT-PCR; **p < 0.01 vs HCCLM3/si-NC.

**Additional file 3: Fig. S2**. Effect of IGFBP2 on the expression of β-catenin in HCCLM3 cells. (A) Expression of β-catenin was detected by western blot. (B) Expression and localization of β-catenin was detected by immunofluorescence. Scale bars, 50 μm. (C) The transcript level of β-catenin stimulated by recombinant human IGFBP2 in HCCLM3 cells was detected by double luciferase reporter gene method. **p < 0.01 vs Ctr. (D) Co-expression of IGFBP2 and LRP6 were detected by immunofluorescence. Scale bars, 50 μm.

## Data Availability

All the data in this study can be provided by the corresponding author if needed.
