# Supplementary material for: IGFBP2 drives epithelial-mesenchymal transition in hepatocellular carcinoma via activating the Wnt/β-catenin pathway
Source: Infect Agent Cancer. 2023 Nov 13;18:73. doi: 10.1186/s13027-023-00543-6 (PMC10644524; doi:10.1186/s13027-023-00543-6)
Supplement: Supplementary file 1 — Additional file 1. Correlation between the IGFBP2 expression and the clinicopathologic features of HCC. Statistical analyses were carried out using Chi-square χ2 test , * represented p < 0.05 was considered significant. [file 13027_2023_543_MOESM1_ESM.pdf]

Table S1 Correlation between the IGFBP2 expression and the clinicopathologic features of HCC

Parameter	Tumor IGFBP2 Expression: Low expression (-/+)	Tumor IGFBP2 Expression: High expression (++ / +++)	χ^2	*P*-value
All cases	23	27		
Age, years			2.339	0.107
>50	7	14		
≤50	16	13		
Sex			0.410	0.362
Male	9	13		
Female	14	14		
Tumor size, cm			6.799	0.0093*
>5	17	10		
≤5	6	17		
Lymph node metastasis			0.008	0.464
Yes	14	15		
No	9	12		
TNM stage			0.027	0.552
I/II	8	10		
III/IV	15	17		
Organ metastasis			7.177	0.008*
Yes	5	16		
No	18	11		

Statistical analyses were carried out using Chi-square $\chi 2$ test. * represented $p<0.05$ was considered significant.
