# Supplementary figures and images for: IGFBP2 drives epithelial-mesenchymal transition in hepatocellular carcinoma via activating the Wnt/β-catenin pathway
Source: Infect Agent Cancer. 2023 Nov 13;18:73. doi: 10.1186/s13027-023-00543-6 (PMC10644524; doi:10.1186/s13027-023-00543-6)

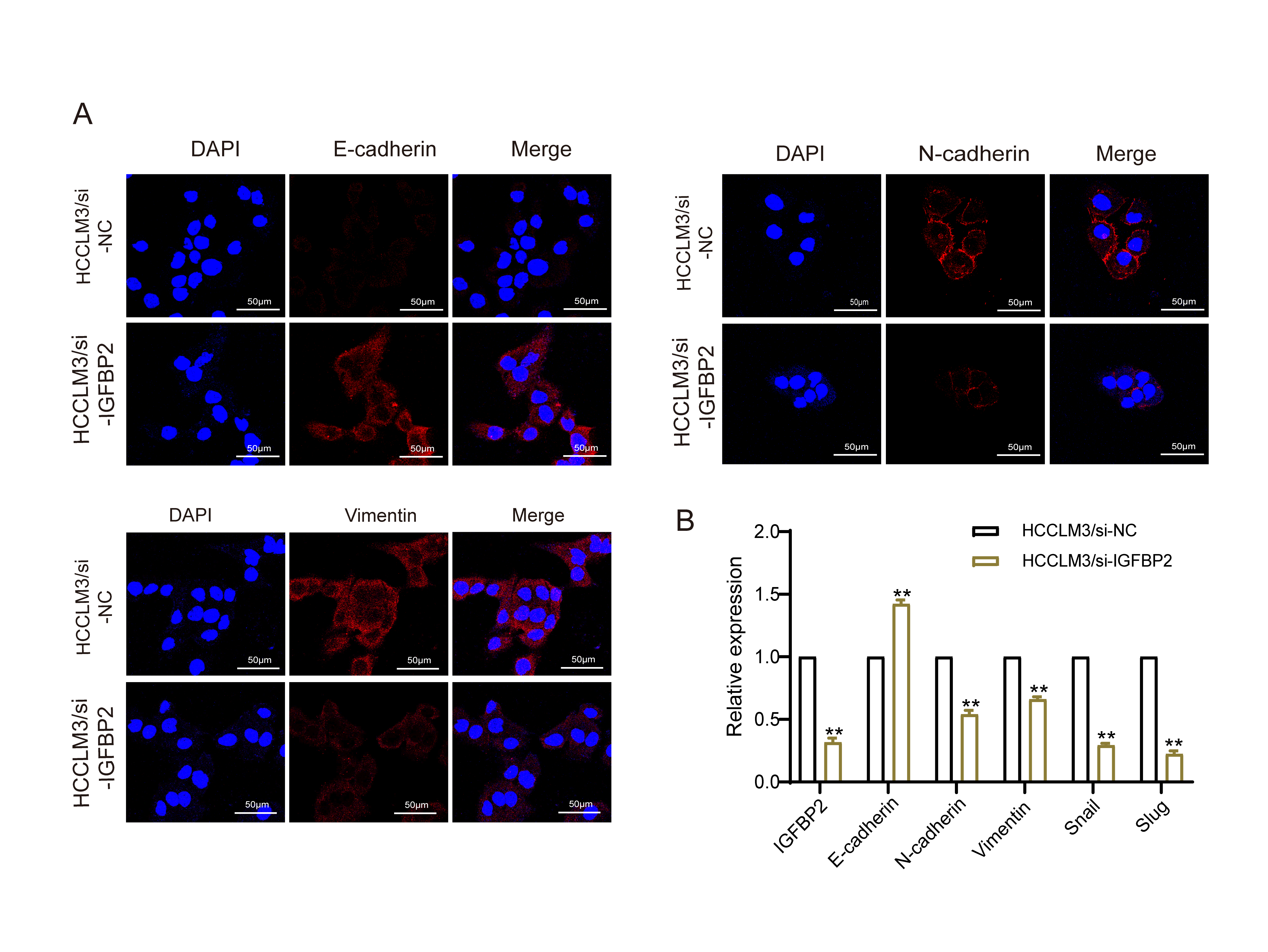

Supplement: Supplementary file 2 — Additional file 2: Fig. S1. Effect of IGFBP2 on the expression of EMT related proteins in HCCLM3 cell were detected by immunofluorescence. Scale bars, 50 μm. (B) Effect of IGFBP2 on the expression of EMT related gens in HCCLM3 cell were detected by RT-PCR; **p < 0.01 vs HCCLM3/si-NC. [file 13027_2023_543_MOESM2_ESM.tif]

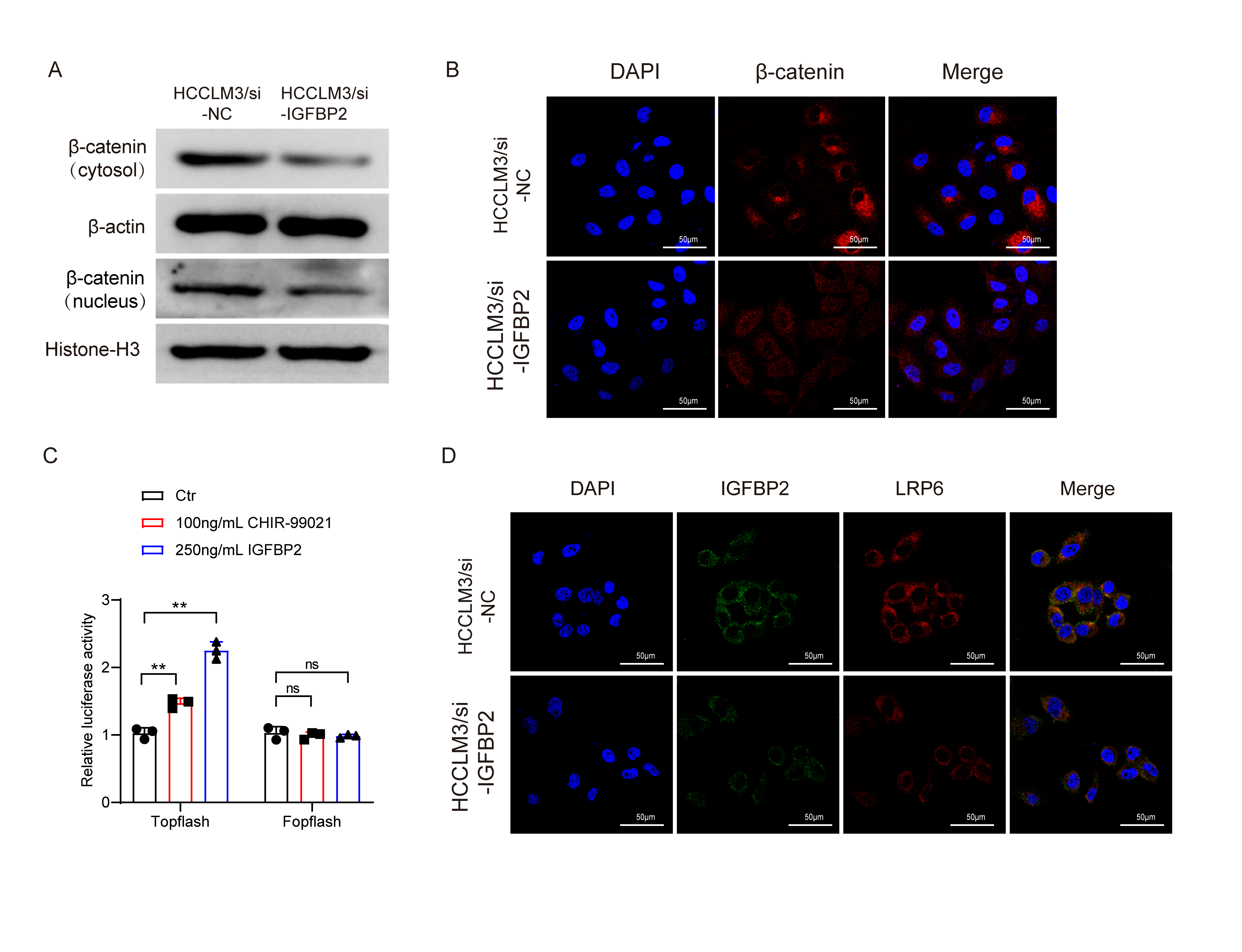

Supplement: Supplementary file 3 — Additional file 3: Fig. S2. Effect of IGFBP2 on the expression of β-catenin in HCCLM3 cells. (A) Expression of β-catenin was detected by western blot. (B) Expression and localization of β-catenin was detected by immunofluorescence. Scale bars, 50 μm. (C) The transcript level of β-catenin stimulated by recombinant human IGFBP2 in HCCLM3 cells was detected by double luciferase reporter gene method. **p < 0.01 vs Ctr. (D) Co-expression of IGFBP2 and LRP6 were detected by immunofluorescence. Scale bars, 50 μm. [file 13027_2023_543_MOESM3_ESM.tif]
